# A cytokine-induced spheroid-based *in vitro* model for studying osteoarthritis pathogenesis

**DOI:** 10.3389/fbioe.2023.1167623

**Published:** 2023-05-09

**Authors:** Annachiara Scalzone, Giorgia Cerqueni, Xiao Nong Wang, Kenny Dalgarno, Monica Mattioli-Belmonte, Ana M. Ferreira-Duarte, Piergiorgio Gentile

**Affiliations:** ^1^ School of Engineering, Newcastle University, Newcastle UponTyne, United Kingdom; ^2^ Center for Advanced Biomaterials for Healthcare@CRIB Istituto Italiano di Tecnologia, Napoli, Italy; ^3^ Department of Clinical and Molecular Sciences (DISCLIMO), Università Politecnica delle Marche, Ancona, Italy; ^4^ Translational and Clinical Research Institute, Newcastle University, Newcastle UponTyne, United Kingdom

**Keywords:** *in vitro* model, articular cartilage, osteoarthritis, cytokines, chondrocytes

## Abstract

Given the lack of *in vitro* models faithfully reproducing the osteoarthritis (OA) disease on-set, this work aimed at manufacturing a reliable and predictive *in vitro* cytokine-based Articular Cartilage (AC) model to study OA progression. Cell spheroids of primary human fetal chondrocytes (FCs) and h-TERT mesenchymal stem cells differentiated chondrocytes (Y201-C) were analysed in terms of growth kinetics, cells proliferation and apoptosis over 10 days of culture, in healthy condition or in presence of cytokines (interleukin-1ß, −6 and TNF-α). Then, the spheroids were assembled into chondrospheres using a bottom-up strategy, to obtain an *in vitro* cytokines-induced OA model. The resulting chondrospheres were evaluated for gene expression and anabolic ECM proteins. Compared to the healthy environment, the simulated OA environment induced chondrocyte hyperproliferation and apoptotic pathway, decreased expression of anabolic ECM proteins, and diminished biosynthetic activity, resembling features of early-stage OA. These characteristics were observed for both Y201-C and HC at high and low concentrations of cytokines. Both HC and Y201-C demonstrated the suitability for the manufacturing of a scaffold-free *in vitro* OA model to facilitate studies into OA pathogenesis and therapeutic strategies. Our approach provides a faithful reproduction of early-stage osteoarthritis, demonstrating the ability of obtaining different disease severity by tuning the concentration of OA-related cytokines. Given the advantages in easy access and more reproducible performance, Y201-C may represent a more favourable source of chondrocytes for establishing more standardized protocols to obtain OA models.

## 1 Introduction

Among different forms of joint diseases, the progressive degeneration of Articular Cartilage (AC) due to osteoarthritis (OA) is the most common and chronic, with a global prevalence of weight-bearing joints such as the hip and knee (Yuan et al., 2003; Cross et al., 2014). OA and the associated burden costs are revealing an increasing impact worldwide, representing a major public health challenge (Kloppenburg & Berenbaum, 2020). Being a “whole joint” disease, OA pathophysiology involves cellular changes, structural defects and dysfunction of all the joint compartments, i.e., cartilage, bone and synovium (Scanzello & Goldring, 2012; Rigoglou & Papavassiliou, 2013). In healthy AC, chondrocytes have an active biosynthetic activity, expressing and synthesizing anabolic markers like collagen type 2 (Coll II) and aggrecan. During OA, chondrocytes tend to both proliferate, possibly due to better access of chondrocytes to proliferative factors from the synovial fluid, caused by damages to the matrix itself, and to undergo an inappropriate hypertrophy-like maturation and become apoptotic (at late OA) (Blanco et al., 1998). Current studies have demonstrated a central role of the inflammatory process, mediated by pro-inflammatory mediators (i.e., cytokines and chemokines) in OA pathogenesis, which are responsible for the production of degradative enzymes (i.e., aggrecanases and collagenases) by chondrocytes while inhibiting their anabolic activity. These enzymes digest the extracellular matrix (ECM), causing aggrecan cleavage and Coll II fibrillation (Valdes, 2010).

Several therapeutic strategies have been exploited for OA treatment, based on the level of pain and degeneration of the diarthrodial joint. Amongst those, regenerative medicine approaches (i.e., autologous chondrocyte implantation, ACI) increased in popularity in the field in the last two decades. Particularly, a variation of ACI, called Chondrosphere^®^ has been clinically approved and recommended by the NICE in 2017 for treating symptomatic AC defect up to 10 cm^2^. Since OA progression is a complex, dynamic, and metabolically active process that involves the balance between anabolic and catabolic processes affecting the joint tissues, there is still a lack of knowledge about the OA evolution process and its internal mechanisms (Gikas et al., 2009).

In this scenario, reliable and predictive *in vitro* models of AC tissues in healthy and OA conditions play a critical role in advancing the understanding of the physiology, biology, and pathological progression of OA, and will contribute hugely to the development of novel regenerative treatments for OA (Grenier et al., 2014; Johnson et al., 2016). A variety of proinflammatory cytokines are commonly used to induce the ECM degradation seen in human OA which involves factors i.e., interleukins (IL-1α, IL-1β, IL-6, IL-8, IL-9) and tumour necrosis factor (TNF-α) (Gabriel et al., 2010; Novakofski et al., 2012; Sylvester et al., 2012). Amongst them IL-1β, TNF-α and IL-6 are the three cytokines mostly induced in OA pathogenesis and possess a synergic effect: (i) IL-1β, which was found to inhibit Coll II and proteoglycans (PGs) and stimulate the production of MMP1,3,13, (ii) IL-6 to activate NF-kB pathway and downregulate the enzymatic antioxidant defenses in chondrocytes with mitochondria; (iii) TNF-α, which can activate the NF-kB pathway, increase the expression of MMPs, inhibit anabolic molecules, and stimulate chondrocytes apoptosis (Fan et al., 2007; Calich et al., 2010; Meliconi et al., 2013; Löfgren et al., 2018). Particularly, lots of studies focused on IL-1β and successfully demonstrated the effect of therapeutic compounds on the OA-induced model; whereas TNF-α and IL-6, which play a main role in the pathophysiology of OA, have only been investigated in a few experimental trials (Bondeson et al., 2010; Goldring & Goldring, 2016). In addition, the concentration of cytokines used for *in vitro* studies up to date is lower than the ones detected in synovial fluid of OA patients, therefore it is hard to obtain a faithful representation of the native OA environment (Bartolotti et al., 2021). Despite several *in vitro* models having been developed and refined over the years, there is still no confirmed gold standard which can be applied when developing OA drug molecules and/or drug delivery systems.

Thus, this work aims to study the OA progression in a multiple cytokines-induced *in vitro* AC model. To this goal, a pathological model is designed by loading the cell media with a cocktail of cytokines including IL-1ß, IL-6 and TNF-α. The use of a combination of cytokines would induce OA-like cell and tissue responses to closely resemble the native disease, particularly taking into consideration the importance of synovium effects in the model design (Johnson et al., 2016). This pathological model was based on a reliable *in vitro* model of healthy AC, developed in our previous work (Scalzone et al., 2022b), whereas cytokines concentration was selected according to their prevalence in OA synovial fluid (Sohn et al., 2012). The main features which distinguish Healthy and OA conditions were analyzed with primary human fetal chondrocytes (FC) and Y201 TERT mesenchymal stem cells differentiated chondrocytes (Y201-C) using a pathological *in vitro* OA model.

## 2 Experimental section

### 2.1 Materials

All the reagents were obtained from Sigma-Aldrich (United Kingdom) unless differently stated. The ultrapure water employed (dH_2_O) throughout the experiments was obtained with a Milli-Q Integral system equipped with a BioPak ultrafiltration cartridge (Millipore, Merck).

### 2.2 Cell culture: FCs and Y201-C

FC was cultured (Cell Application, United States) in ready-to-use Chondrocyte Growth Medium (PromoCell, United Kingdom) at 37°C in a humidified atmosphere incubator containing 5% CO_2_. At 80% confluence, cells were detached with trypsin-EDTA (PAA-Laboratories GmbH, Germany) and subcultured to passage 6 for experiments. Human TERT immortalised bone marrow stromal cells (Y201) were supplied by Prof P. Genever (York University) and cultured at 37°C and 5% CO_2_ in Dulbecco’s Modified Eagle Medium (DMEM) with low glucose content, supplemented with 10% Foetal bovine serum (FBS), 2 mM L-glutamine and a 1% Penicillin/Streptomycin (P/S). After the expansion, cells were differentiated in chondrocytes (Y201-C), as previously reported (Scalzone et al., 2022b).

### 2.3 Manufacturing of *in vitro* OA model

Spheroids were formed as previously reported in our work (Scalzone et al., 2023). Briefly, FC (passage 6) and Y201-C (passage 15) were seeded in a round bottom non-tissue treated 96-well plate (ThermoFisher Scientific, United Kingdom) at a density of 2 × 10^5^ cells/well, suspended in 150 µL of DMEM/F12 supplemented with 0.25% (w/v) methylcellulose (MC). Then, the multi-well plates were incubated at 37°C and 5% CO_2_ for 10 days. After 1 day of culture, three different conditions were set for the spheroids, as reported in Table 1, and the media was changed every 2 days.

**TABLE 1:** Culture conditions for FC and Y201-C: High concentration of cytokines (OA-HC), Low concentration of cytokines (OA-LC) and Healthy.

Cytokines	High concentration (OA-HC)	Low concentration (OA-LC)	Healthy
**IL-1β**	5 ng/mL	1 ng/mL	-
**TNF-α**	5 ng/mL	1 ng/mL	-
**IL-6**	50 ng/mL	10 ng/mL	-

**FIGURE 1:**
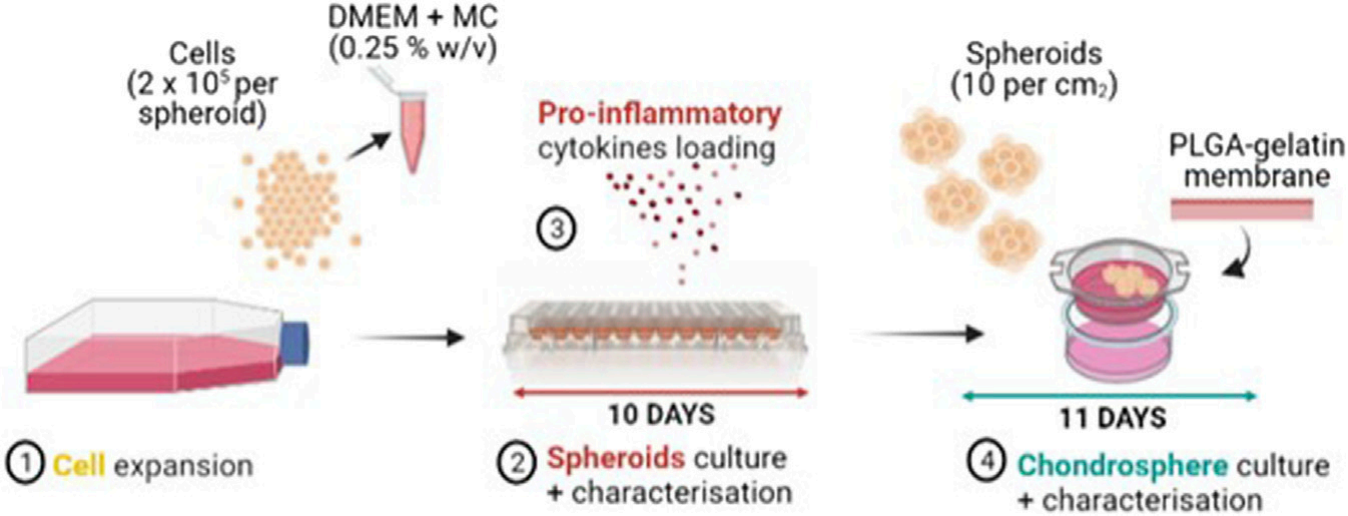
Manufacturing process of the OA *in vitro* model: FC and Y201-C cells expansion (1), Spheroids culture and characterization in a round bottom 96-well plate for 10 days (2) with the addition of IL-6, IL-1ß, TNF-α proinflammatory mediators at day 3 of spheroids culture (3), Chondrospheres culture and characterisation for further 11 days on a gelatin-coated electrospun PLGA membrane (4).

Following a procedure optimised in our previous work, after 10 days of spheroids culture, five single spheroids were transferred onto an electrospun poly(lactic-co-glycolic acid) (PLGA)-dopamine functionalised and gelatin-coated membranes, that were mounted in 48-multiwell CellCrowns™ (10 spheroids per cm^2^ of membrane) to obtain chondrospheres, which were cultured in DMEM/F12 medium for further 11 days at 37°C in a humified atmosphere with 5% CO_2_. The whole process for manufacturing the *in vitro* OA model is reported in Figure 1.

### 2.4 Spheroids growth kinetics during the culture

Spheroid growth kinetics was monitored over 10 days of culture for all conditions: Healthy, OA-HC and OA-LC. Images were taken on days 1, 4 and 10 with an EVOS M5000 microscope in phase-contrast brightfield. Spheroids morphometric analysis was performed by measuring diameters and areas of at least 4 spheroids for time points using ImageJ software. Results were analysed using GraphPad Prism software (GraphPad Software, Inc., La Jolla, CA).

### 2.5 Metabolic activity assessment

To evaluate cells metabolic activity MTS (3-(4,5-dimethylthiazol-2-yl)-5-(3-carboxymethoxyphenyl)-2-(4-sulfophenyl-2H-tetrazolium) assay (CellTiter 96^®^ AQueous One Solution Cell Proliferation Assay, Promega, United Kingdom) was used. Each condition was analysed in triplicate on days 1, 4 and 10. MTS solution was prepared to dilute at 1:6 the CellTiter 96^®^ AQueous One Solution Reagent in phenol red-free DMEM/F12 supplemented with 10% FBS and 1% P/S. 200 μL of MTS solution was added to each well that contained the spheroids and incubated at 37°C in a humidified 5% CO_2_ atmosphere for 2.5 h and then 90 μL of the solution was transferred into a 96-well plate in duplicate for each sample. Absorbance was recorded at 490 nm using a Filter-based FLUOstar^®^ Omega multi-mode reader (FLUOstar^®^ Omega, Germany). The results were analysed using GraphPad Prism 9 software.

### 2.6 Gene expression analysis

For gene expression analysis, total RNA was extracted from spheroid and chondrosphere samples using miRNeasy Micro RNA Isolation Kit (Qiagen, United States), according to the manufacturer’s protocol. The concentration and purity of the isolated RNA were measured using a spectrophotometer (NanoDrop™ 1,000, Thermo Fisher Scientific, United States). Reverse transcription was performed using the High-Capacity cDNA Reverse Transcription Kit (Thermo Fisher, United Kingdom), using a thermocycler (2,720 Thermal Cycler, Applied Biosystems, United States) based on cycles of 10 min at 25°C, 120 min at 37°C, 5 min at 85°C. Reverse Transcription quantitative real-time Polymerase Chain Reaction (RT-qPCR) was performed using TaqMan™ Fast Advanced Master Mix and TaqMan™ *SOX9, ACAN, COL2A1, COL1A2, MMP13* and *ADAMTS5* probes (Thermo Fisher Scientific, United Kingdom). Gene expression raw data were acquired using QuantStudio™ 3 Real-Time PCR System (Thermo Fisher Scientific, United States). The expression of the genes of interest was normalized to Glyceraldehyde-3-phosphate dehydrogenase (*GAPDH)* and presented as a relative expression using the 2^−(ΔΔCt)^ method(Livak & Schmittgen, 2001). The expression levels of day 0 control conditions for FC and Y201-C were set to 1 then the expression levels of day 10 and day 21 conditions were presented as fold-change relative to the controls. The ratio of COL2A1/COL1A2 was evaluated as well on day 10 and day 21.

### 2.7 Histology and immunohistochemistry (IHC) assessment

Samples of both Y201-C and FC in all conditions on days 1, 7 and 21 were washed with PBS, moved to a 0.5 mL Eppendorf embedded in OCT Compound (Agar Scientific, United Kingdom) and snap frozen at −80°C until further use. Samples were cryosectioned using a CM1900 cryostat (Leica Biosystems, Germany) at −20°C at 5 μm thickness, let dry at room temperature for 2 h and kept frozen until analysis. To perform Histology and IHC, frozen slides were transferred to cold Acetone at −20°C for 10 min. Following this, the fixative was let evaporate for 15–20 min and the slides were washed twice with PBS to remove any trace of OCT compound.

Hematoxylin and Eosin (H&E), Picrosirius Red and Alcian Blue stainings were performed according to standard procedures (Scalzone et al., 2022a). Histology slides were let dry overnight under the fume hood and imaged the following day with EVOS M5000 Microscope in RGD Brightfield at × 20 and × 40 magnification. For IHC, fixed and washed tissue slices were permeabilised with Triton 100x solution (0.1% w/v in PBS), immersed in BSA (2% w/v in PBS) and incubated for 2 h at RT with primary antibodies rabbit Anti-Collagen II (ab34712 Abcam) or rabbit Anti-Ki-67 (NB500-170SS Bio-techne Ltd.) or mouse Anti-Aggrecan (ab3778 Abcam) respectively at 1:200, 1:100 and 1:50 in BSA solution. After washing in PBS, secondary antibodies solutions were added for 1 h: AlexaFluor-594 goat anti-rabbit IgG (H + L) (ab150080 Abcam) 1:500 in BSA solution to Anti-Collagen II slides, Fluorescein-labelled goat anti-rabbit IgG (H + L) (F2765, Thermo Fisher Scientific) 1:1,000 in BSA solution for Anti-Ki-67 and Goat anti-Mouse IgG (H&L) - Alexa Fluor™ 488 (A-11001, Thermo Fisher Scientific) 1:1,000 in BSA solution for Anti-Aggrecan slides. Finally, nuclei were counterstained with Hoechst solution (R37609 Thermo Fisher Scientific), according to the manufacturer’s instructions, and a drop of Invitrogen ProLong Glass Antifade Mountant (Fisher Scientific, United Kingdom) was added to each slide and covered with a rectangular coverslip. Slides were let dry under the hood for 15 min and imaged using EVOS M5000 Microscope in fluorescence at × 40 magnification.

### 2.8 Statistical analysis

Tests were performed at least in triplicate for each sample. The results were represented as mean ± standard deviation. Differences between groups were determined using a One-way analysis of variance (ANOVA) with Tukey’s multiple comparison test using levels of statistical significance of *p* < 0.0001 (****), *p* < 0.001 (***), *p* < 0.05(**) and *p* < 0.01(*).

## 3 Results

### 3.1 Spheroids growth kinetics and metabolic activity

**FIGURE 2:**
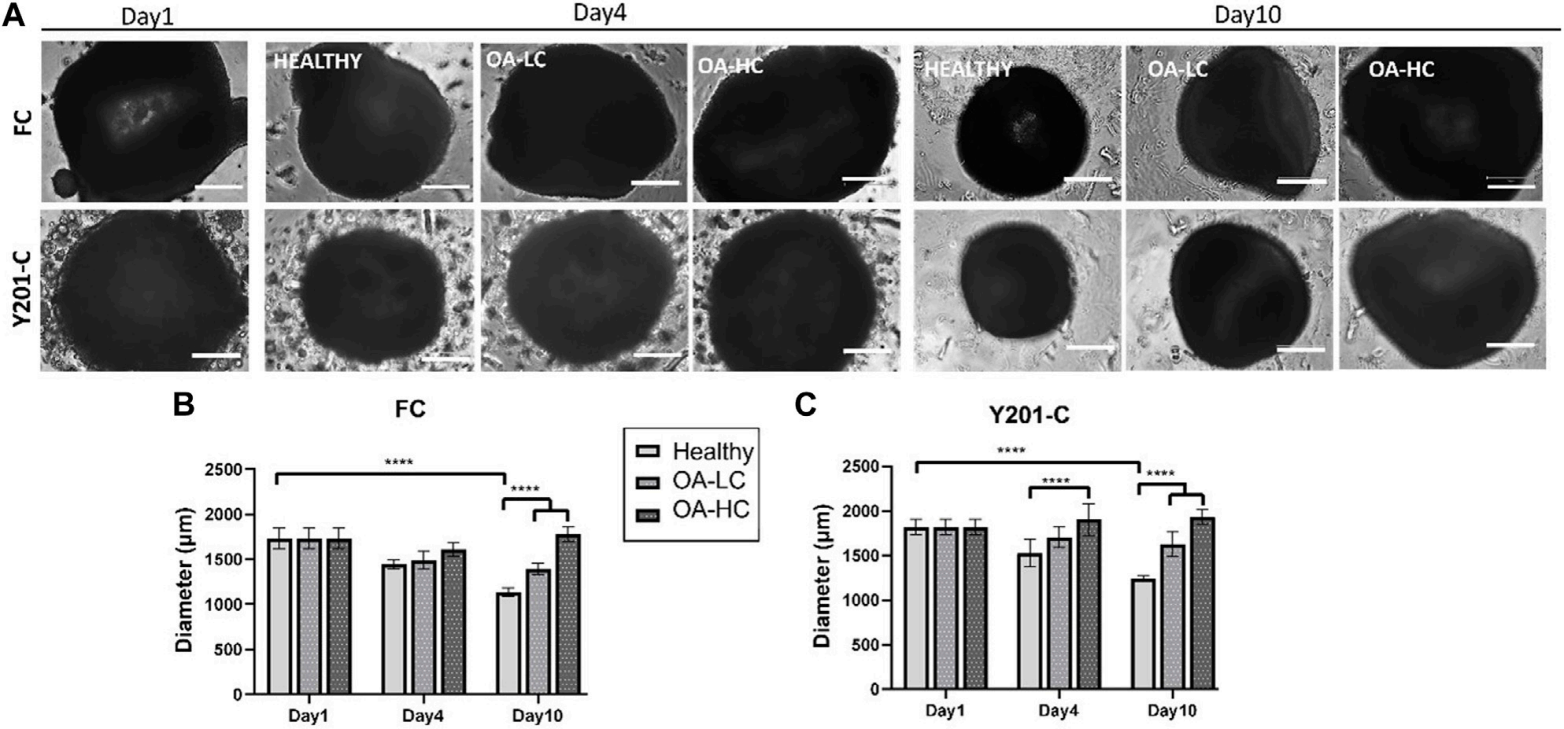
FC and Y201-C spheroids images on day 1, day 4 and day 10 in Healthy, OA-LC and OA-HC conditions. Scale bars:300 µm. **(A)** Spheroids diameter at different timepoints for FC **(B)** and Y201-C **(C)** at each condition. Statistics: ****p < 0.0001.

Spheroids growth dynamics were analysed on days 1, 4 and 10 for both FC and Y201-C cells in Healthy, OA-LC and OA-HC conditions. From the images collected, it was noticed a gradual decrease in both healthy spheroids’ size (Figure 2A), and a significant decrease in their diameter’s dimension (Figures 2B, C). Particularly, the diameter of the FC spheroids significantly decreased from 1,610 ± 100 µm on day 1–1,005 ± 90 µm on day 10 (*p* < 0.0001), while Y201-C spheroid diameter significantly decreased from 1750 ± 90 µm on day 1–1,200 ± 70 μm at day 10 (*p* < 0.0001). In the OA spheroids condition, a different trend was observed for both FC and Y201-C, with a stable dimension of spheroids over the culture at low and high concentrations of cytokines. On day 10, FC spheroids achieved values of 1,450 ± 50 μm and 1,650 ± 50 µm for OA-LC and OA-HC samples, respectively. These values were significantly different (*p* < 0.0001) compared to the Healthy FC on day 10. Similarly, Y201-C spheroids in OA-LC and OA-HC conditions showed values of 1,600 ± 50 μm and 1850 ± 50 μm, respectively, significantly different (*p* < 0.0001) from Y201-C in Healthy conditions on day 10.

**FIGURE 3:**
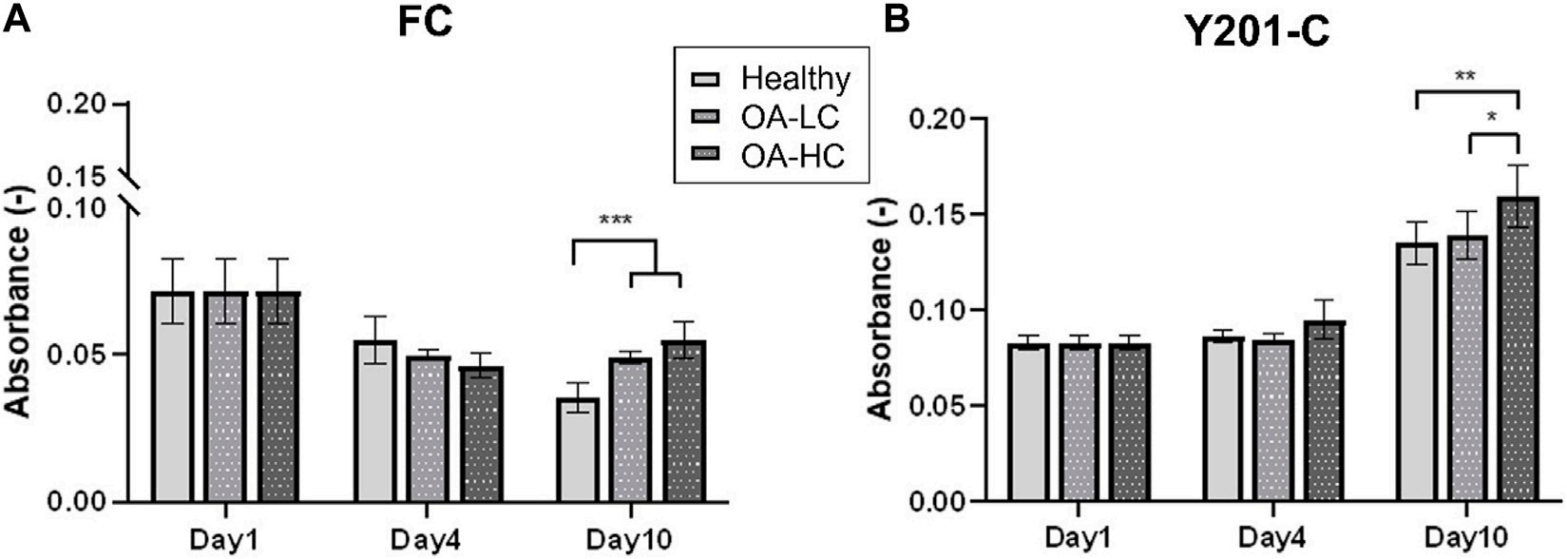
Analyses of cellular mitochondrial activity in Healthy, OA-LC and OA-HC conditions, for FC **(A)** and Y201-C **(B)**. Statistics: **p < 0.05 and *p < 0.01.

Cellular mitochondrial activity was estimated via MTS assay (Figure 3). FC spheroids metabolic activity decreased over culture (*p* < 0.0001) in all conditions: from 0.07 ± 0.03 on day 1 to 0.03 ± 0.02 in Healthy, 0.04 ± 0.01 in OA-LC and 0.05 ± 0.02 in OA-HC conditions at day 10. Y201-C spheroids metabolic activity showed a different trend, increasing statistically (*p* < 0.0001) from 0.08 ± 0.01 on day 1 to 0.13 ± 0.05, 0.14 ± 0.05 and 0.16 ± 0.07, respectively in Healthy, OA-LC and OA-HC conditions at day 10. Both FC and Y201-C showed higher metabolic activity for OA-HC samples compared to OA-LC (*p* < 0.01) and Healthy (*p* < 0.05) (Figures 3A, B).

### 3.2 Analyses of cell proliferation and apoptotic tendency

**FIGURE 4:**
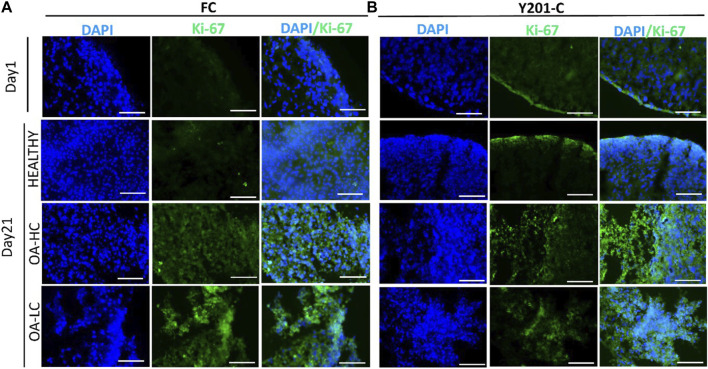
Assessment of cellular proliferation on day 1 and day 21 in Healthy, OA-LC and OA-HC conditions with Ki-67 (green) staining and nuclei counterstained with DAPI (blue), for FC **(A)** and Y201-C **(B)**. Scale bars: 150 µm.

Proliferation and apoptosis were evaluated on cryosections on day 1 and day 21. Cellular proliferation was assessed via immunostaining for Ki-67 (an antigen associated with cellular proliferation) and cell nuclei were counterstained for DAPI (blue) (Figure 4). A brighter signal corresponding to Ki-67 staining was observed in both FC (Figure 4A) and Y201-C (Figure 4B) in OA conditions (both in OA-HC and OA-LC) on day 21, compared to the Healthy control on day 21 and compared to day 1 control. While few cells in a proliferative state were found on the construct edges in the Y201-C on days 1 and 21 in healthy conditions, compared to the FC.

**FIGURE 5:**
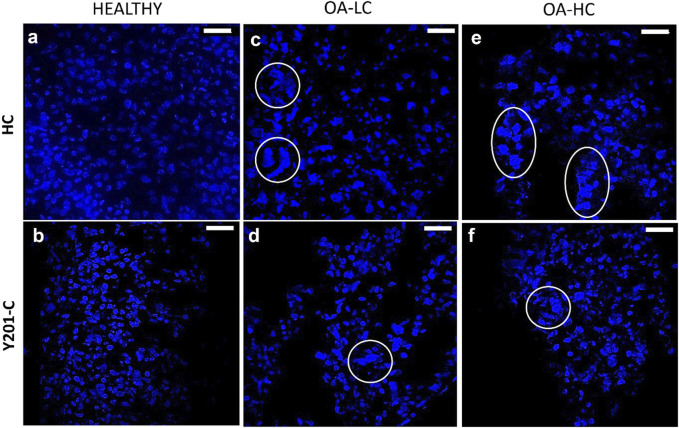
Assessment of cells nuclei organisation and morphology at 21 days in Healthy **(A,B)**, OA-LC **(C,D)** and OA-HC **(E,F)** conditions with DAPI (blue) staining at × 63 magnification. Circles are in correspondence with cellular clusters. Scale bars: 20 µm.

Chondrocytes’ apoptotic features, typical of late-stage OA were analysed on cryosections at 21 days at high magnification (×63) staining cells nuclei with DAPI (Figure 5). Both, FC and Y201-C in a healthy state, showed typical rounded-shaped cell nuclei homogenously dispersed within the tissue slices (Figures 5A, B). While in OA condition (OA-LC and OA-HC), both cells showed heterogeneous nucleic acids organisation toward clustering structures (Figures 5C–F, circles). Also, in the OA condition, cells had fragmented and pyknotic nuclei.

### 3.3 Gene expression analysis

**FIGURE 6:**
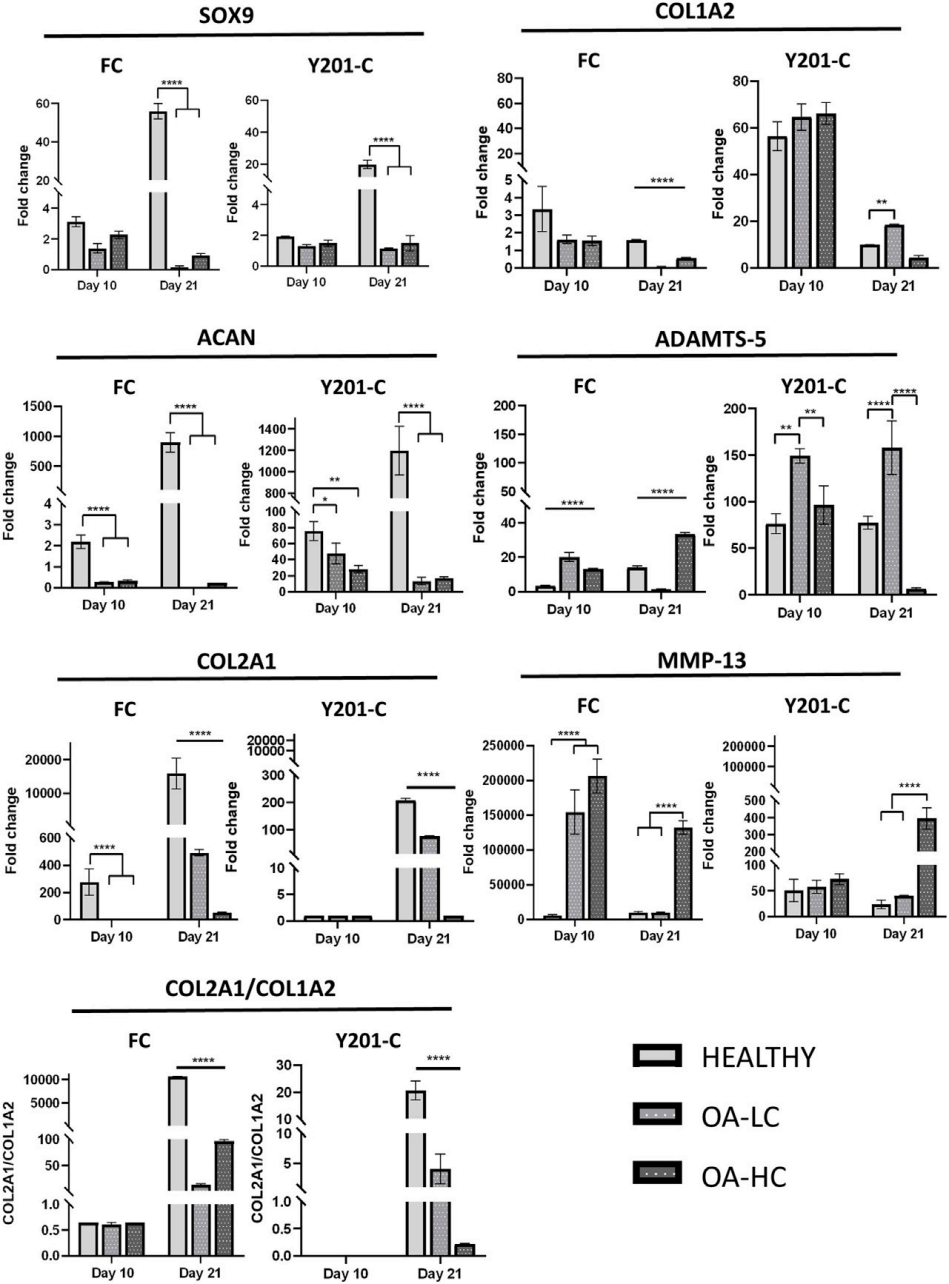
Gene expression analysis via RT-qPCR, for both FC and Y201-C in Healthy, OA-LC and OA-HC: SOX-9, COL1A2, ACAN, ADAMTS-5, COL2A1, MMP-13 and report of the col2a1/col1a2 ratio for FC and Y201-C. Statistics: ****p < 0.0001, **p < 0.01, *p < 0.05.

The expression of anabolic (*SOX9, COL2A1, ACAN* and *COL1A2*) and Catabolic (*MMP13* and *ADAMTS-5*) markers was analysed and reported in Figure 6. Both FC and Y201-C showed a significant increase in *SOX9, ACAN* and *COL2A1* gene expression over the 21 days of culture in healthy conditions. *SOX9* and *ACAN* showed an upregulation at 21 days for both cells in Healthy conditions compared to the relative OA-HC and OA-LC conditions (*p* < 0.0001). Similarly, a significant difference in the *COL2A1* expression between the three different conditions at 21 days for both FC and Y201-C can be observed, with a gradual increase of the expression from OA-HC to Healthy. *COL1A2* displayed an upregulation on day 10, followed by a significant decrease in its expression in each condition for both cells from day 10 to day 21. Also, Healthy FC showed a higher expression of *COL1A2* compared to the pathological, while Y201-C showed the highest fold-regulation in the OA-LC condition. *MMP13* catabolic marker expression increased from day 1 to day 10 for the FC in all conditions and then, decreased after 21 days (Figure 6E): OA-HC showed a much higher expression on both day 10 and day 21, compared to Healthy and OA-LC. For Y201-C, instead, a similar trend was observed for OA-LC and Healthy condition, with an increase of *MMP13* expression on day 10 and a decrease on day 21, while *MMP13* expression significantly increased over culture in the case of OA-HC, showing values significantly higher compared to Healthy and OA-LC (*p* < 0.0001). Finally, *ADAMTS-5* catabolic marker expression increased on day 10 for both cell types in each condition, with significant differences observed on day 21. FC showed a similar value of OA-LC and Healthy on day 21 compared to day 10, while they presented a decrease in *ADAMTS-5* expression. All values were significantly different (*p* < 0.0001) from each other at 21 days, with OA-LC presenting the highest expression. On the other side, Y201-C showed an increase in *ADAMTS-5* expression on day 21 in healthy and OA-HC conditions and a decrease in OA-LC compared to day 10. All values were significantly different (*p* < 0.0001) from each other at 21 days, with OA-HC presenting the highest expression. In addition, the ratio of *COL2A1/COL1A2* did not differ between the three conditions after 10 days of culture (∼0.65 for FC and ∼0 for Y201-C), while it showed statistically significant differences at day 21 with values of: 10,614.0 ± 32.7 in healthy condition compared to 12.3 ± 2.5 and 96.4 ± 3.2 in LC-OA and HC-OA for FC, respectively; and 20.66 ± 3.5 in healthy samples compared to. 4.1 ± 2.5 and 0.2 ± 0.1 in LC-OA and HC-OA for Y201-C, respectively.

### 3.4 Analysis of the quality of tissue in the healthy and pathological state

**FIGURE 7:**
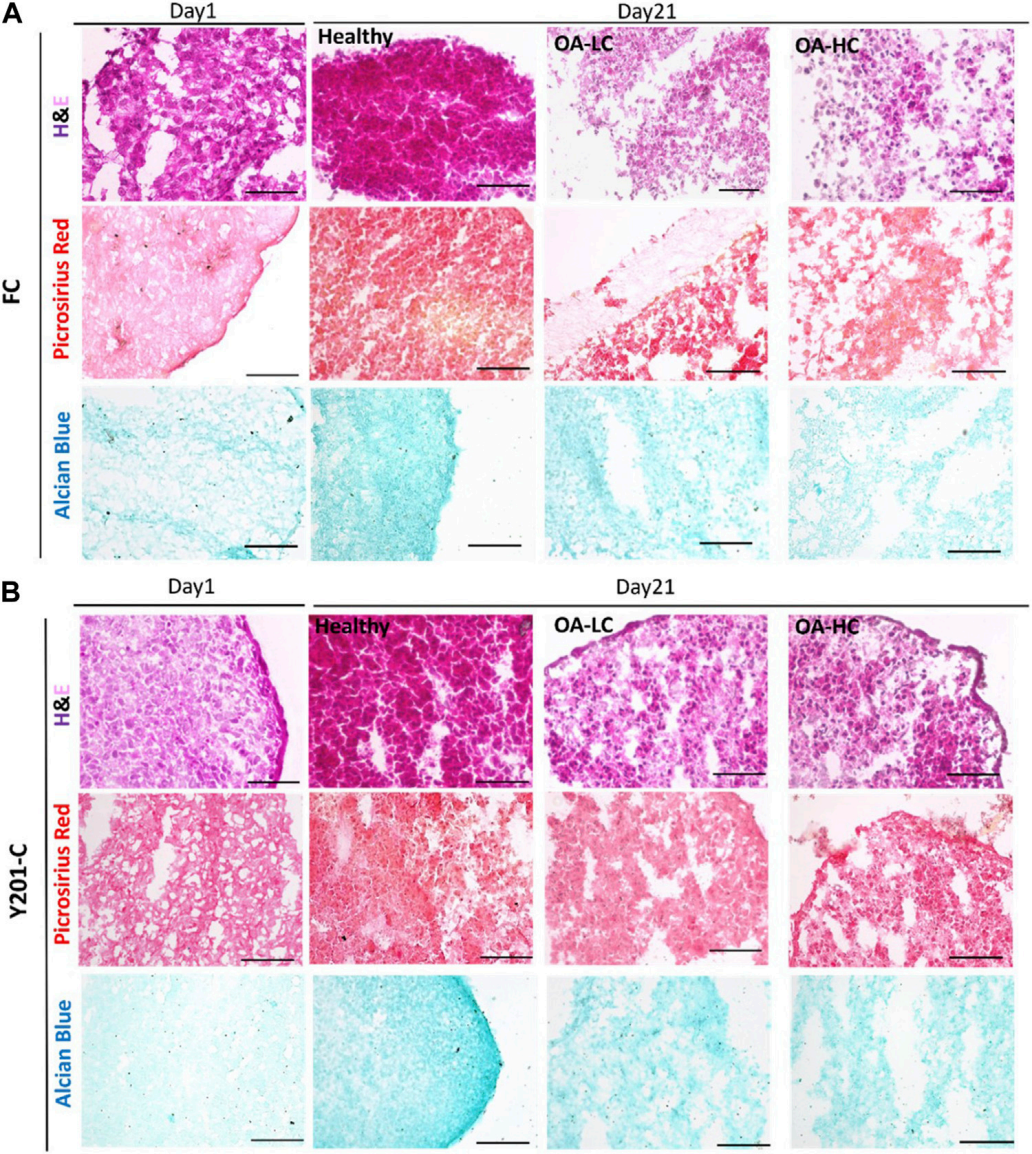
Histological analyses on cryosections at day 1 and day 21 in Healthy, OA-LC and OA-HC conditions. H&E, Picrosirius Red and Alcian Blue stainings for FC **(A)** and Y201-C **(B)** Scale bars = 150 μm.

H&E staining intensity was higher for both FC and Y201-C on day 21 compared to day 1 in Healthy conditions, whereas it was observed a decrease in the staining intensity for OA-LC and OA-HC conditions compared to the healthy control on day 21 (Figures 7A, B). Also, chondrospheres in OA conditions showed a decrease in cellularity for both cell types and this feature seemed to be more evident in the middle of the construct, compared to the edges, especially for the Y201-C cells (Figure 7B). In healthy conditions, both FC and Y201-C samples presented higher collagen deposition (detected with Picrosirius Red) and mucopolysaccharides (detected via Alcian Blue) on day 21, compared to day 1 and both OA-HC and OA-LC on day 21. Chondrospheres cultivated in healthy conditions showed a homogenous distribution of Picrosirius Red and Alcian blue staining, whereas those in OA conditions showed a weaker signal in a restricted area, in the zones of the slices where cells’ nuclei were aggregated.

**FIGURE 8:**
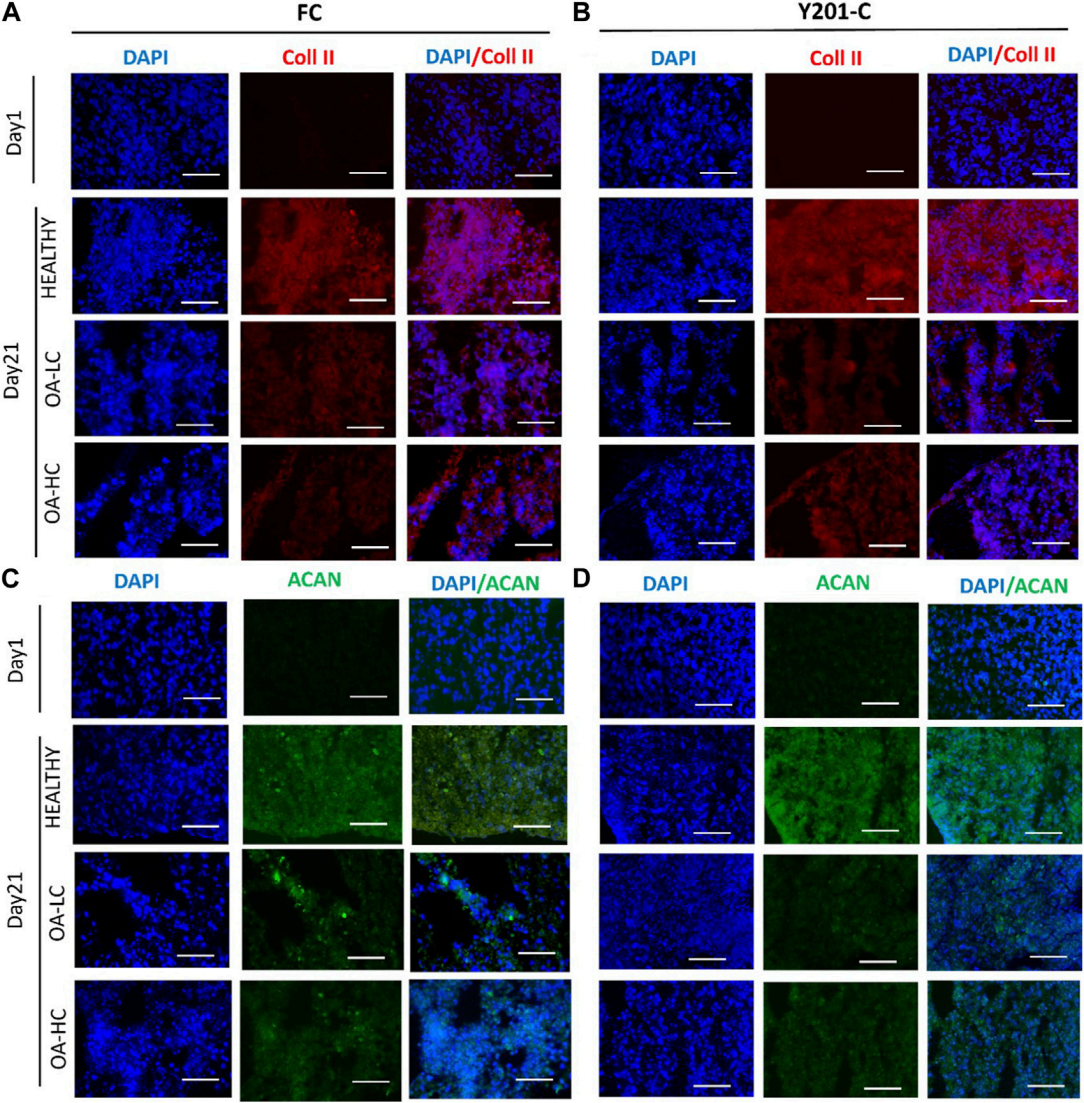
Immunohistochemical analyses of cartilage-specific markers: Collagen type II (Coll II, Red) and Aggrecan (ACAN, Green). Nuclei were counterstained with DAPI (blue). Images were reported in Healthy, OA-HC and OA-LC conditions for Coll II and DAPI for FC **(A)** and Y201-C **(B)**; ACAN and DAPI for FC **(C)** and Y201-C **(D)**. Scale bars = 150 μm.

From the IHC analyses, chondrospheres showed at 21 days an increase in Coll II and Aggrecan staining in healthy conditions, compared to both the control at day 1 and OA conditions at day 21, for both FC and Y201-C. In OA-HC and OA-LC conditions, samples showed less intense staining of Coll II and Aggrecan (Figure 8).

## 4 Discussion

The development of a patho-physiologically relevant *in vitro* model for OA should reflect the complexity of the disease, characterised by AC degeneration accompanied by the whole joint inflammation (Goldberg & Kresina, 1987). Herein, we obtained an OA-induced environment by adding to the culture media the main pro-inflammatory cytokines, released from the synovium cells and chondrocytes, during OA progression (IL-1β, TNFα and IL-6) to recreate a naturally diseased environment (Scalzone et al., 2023). Compared to previous literature, the combination of a cocktail of cytokines allowed to obtain OA-like cell and tissue responses that more closely resemble the native disease at an early stage, particularly taking into consideration the importance of synovium-related inflammation effects in the model design, which results in the release of pro-inflammatory mediators (Johnson et al., 2016).

For both FC and Y201-C, the size of healthy spheroids gradually decreased over 10 days of culture, as dispersed cells formed tightly packed aggregates due to cell-cell interaction and condensation. However, spheroids did not reduce in size when exposed to cytokines, possibly due to a cell proliferation effect as a typical feature of early-stage OA chondrocytes and the interference of cytokines on cell-cell interaction (Gao et al., 2014; Wojdasiewicz et al., 2014). Indeed, a significant difference was observed on day 10 between both pathological conditions and healthy for FC and Y201-C.

Also, Y201-C showed an increase in metabolic activity over culture while FC showed a decrease. However, on day 10, both cells showed a similar pattern: increased metabolic activity in presence of cytokines, which may be related to the production of inflammatory and degradative enzymes, as well as an attempt to repair the ECM that had begun to break down, a characteristic of early-stage OA (Goldring & Goldring, 2016).

Three key events that characterize OA in an early-phase are cell hyperproliferation in contrast to well-established increased production and breakdown of ECM components and inflammation (Quinn et al., 2005; Elmore, 2007). Both cell types showed strong staining of Ki-67, which is a cells proliferation marker, in OA-HC and OA-LC conditions compared to the healthy model on day 21, and the control on day 1 (Miller et al., 2018). This result indicates that cells in pathological conditions tend to proliferate; indeed, OA-induced cells represent a cell population with abnormal proliferation, which is the common chondrocyte response to altered joint environments (Sandell & Aigner, 2001; Dreier, 2010).

Then, it was evaluated at high magnification the morphology of cells nuclei stained with DAPI: both cells in OA conditions, compared to the healthy, showed a decrease in the size of nuclei, their clusterisation, and an anomalous shape due to the presence of apoptotic bodies, nuclear condensation, and fragmentation. All these events are related to the shift of cells towards the hypertrophic pathway, which may result in chondrocyte apoptosis and, as a result, AC loss (Blanco et al., 1998; Muldrew et al., 2001; Elmore, 2007). Also, gene expression was altered in OA conditions: anabolic markers (*SOX9, ACAN* and *COL2A1*) were up-regulated at 21 days of culture in the healthy model with FC and Y201-C compared to both pathological, in which their expression decreased over culture, as expected.

To evaluate the gene expression pathways involved into spheroids weakness in OA environment we compared the expression of *MMP13*, which is the main collagenase responsible for the degradation and cleavage of collagen II, with *COL2A1*. *COL2A1* expression was down-regulated in OA conditions respect Heathy environment while *MMP-13* was massively up-regulated especially in OA-HC for both cell types. Moreover, the expression of *ADAMTS-5*, which together with the *ADAMTS-4* constitutes the family of aggrecanases responsible for the *ACAN* cleavage, was compared with *ACAN*. Also in this context the anabolic and the catabolic markers were inversely related and showed a higher *ACAN* expression in Healthy condition and an *ADAMTS-5* upregulation for both OA-LC and OA-HC. *ADAMTS-5* expression even in the Healthy was in according to literature that confirms that this aggrecanase is constitutively expressed by chondrocytes. The analysis of *COL1A2* by itself does not give great information about the OA progression, but the ratio of *COL2A1/COL1A2*, which was shown to be considerably greater in healthy conditions compared to pathological for both cells, is a crucial indicator between healthy and OA cartilage. However, *COL2A1/COL1A2* ratio decreased mostly due to a change or down-regulation of *COL2A1* expression, whereas *COL1A2* expression altered far less. This characteristic is consistent with earlier literature investigations (Marlovits et al., 2004).

The morphological quality of the spheroid model as the degree of degradation was determined by histology and IHC stainings. H&E staining indicates the presence of nucleated cells with abundant cytoplasmic content in all conditions. The main difference between the healthy and both pathological models is the weaker staining of collagen (stained with Picrosirius Red) and of strongly and weakly sulphated proteoglycans (stained with Alcian Blue) in OA conditions. Also, OA samples showed less compact and more scattered tissue. IHC supported the findings of histology and gene expression: when compared to pathological conditions, FC and Y201-C cells in healthy conditions formed a tissue rich in Coll II and aggrecan, as seen by the stronger staining and the compactness of the tissue slice. This is the result of the inhibition of the biosynthetic activity and matrix degradation by proteinases (Matyas et al., 2002; Roughley & Mort, 2014).

Overall, both cells at 21 days in the pathological state (OA-LC & OA-HC) showed a down-regulation in the expression of anabolic markers, compared to the healthy state and a higher expression of catabolic markers in the pathological model with a high concentration of cytokines.

## 5 Conclusion

Current OA models often replicate either post-traumatic and/or late-stage OA, leaving a large gap in understanding the spontaneous occurring disease and its early stages, where slowing disease progression would be an attractive treatment strategy. The presented *in vitro* model of AC in healthy and pathological condition allowed the study of the OA progression from early stage by demonstrating: (1) stimulated cell proliferation and apoptotic pathway, (2) decreased expression of anabolic markers and (3) lower biosynthetic activity in the simulated pathological environment when compared to the healthy environment. These functional biological changes in the spheroid models were observed even at low cytokine concentrations (in agreement with values seen in native human synovium during OA progression. Furthermore, Y201-C was shown to be a suitable cell source for easier and reproducible manufacturing of scaffold-free *in vitro* OA models. Given the obtained features, this model can be faithfully be used to (i) study OA molecular evolution from the on-set to its degeneration and (ii) to assess the effect and optimize the therapeutic dose of drugs and molecules, such as micro-RNAs or exosomes, targeting for example, the cellular pathways involved in the OA progression.

## Data Availability

The original contributions presented in the study are included in the article/Supplementary Material, further inquiries can be directed to the corresponding author.
